# Transmissibility of acute haemorrhagic conjunctivitis in small-scale outbreaks in Hunan Province, China

**DOI:** 10.1038/s41598-019-56850-9

**Published:** 2020-01-10

**Authors:** Siyu Zhang, Qingqing Hu, Zhihong Deng, Shixiong Hu, Fuqiang Liu, Shanshan Yu, Ruoyun Liu, Chunlei He, Hongye Li, Lidong Gao, Tianmu Chen

**Affiliations:** 1 Hunan Provincial Center for Disease Control and Prevention, 450 Middle Furong Road section 1, Changsha, 410005 Hunan People’s Republic of China; 2 Division of Public Health, School of Medicine, University of Utah, 201 Presidents Circle, Salt Lake City, 84112 Utah USA; 3 State Key Laboratory of Molecular Vaccinology and Molecular Diagnostics, School of Public Health, Xiamen University, Xiamen, Fujian Province People’s Republic of China

**Keywords:** Infectious diseases, Epidemiology

## Abstract

Acute haemorrhagic conjunctivitis (AHC) outbreaks are reported frequently in China. However, the transmissibility of AHC remains unclear. This study aimed to calculate the transmissibility of the disease with and without interventions. An AHC outbreak dataset from January 2007 to December 2016 in different schools was built in Hunan Province. A Susceptible-Infectious-Recovered (SIR) model was adopted to calculate the effective reproduction number (*R*_*eff*_) of AHC. *R*_*eff*_ was divided into two parts (*R*_*unc*_ and *R*_*con*_) where *R*_*unc*_ and *R*_*con*_ represent the uncontrolled and controlled *R*_*eff*_ , respectively. Based on *R*_*unc*_ and *R*_*con*_, an index of effectiveness of countermeasures (*I*_*eff*_) was developed to assess the effectiveness of countermeasures in each outbreak. During the study period, 34 AHC outbreaks were reported in 20 counties of 9 cities in Hunan Province, with a mean total attack rate of 7.04% (95% CI: 4.97–9.11%). The mean *R*_*unc*_ of AHC outbreaks was 8.28 (95% CI: 6.46–10.11). No significance of *R*_*unc*_ was observed between rural and urban areas (*t* = −1.296, *P* = 0.205), among college, secondary, and primary schools (*F* = 0.890, *P* = 0.459), different levels of school population (*F* = 0.738, *P* = 0.538), and different number of index cases (*F* = 1.749, *P* = 0.180). The most commonly implemented countermeasures were case isolation, treatment, and health education, followed by environment disinfection, symptom surveillance, and school closure. Social distance, prophylaxis, and stopping eye exercises temporary were implemented occasionally. The mean value of *R*_*con*_ was 0.16 (range: 0.00–1.50). The mean value of *I*_*eff*_ was 97.16% (range: 71.44–100.00%). The transmissibility of AHC is high in small-scale outbreaks in China. Case isolation, treatment, and health education are the common countermeasures for controlling the disease.

## Introduction

Acute hemorrhagic conjunctivitis (AHC), an infection mostly caused by enterovirus 70 (EV70) and a variant of coxsackievirus A24 (CA24v)^1^, is a rapidly progressive and highly contagious viral disease^2^. The disease was first reported in Ghana, Africa, in 1969 and subsequently spread to several other countries^2–7^. The first outbreak of AHC in China was reported in Hong Kong in 1971^8^. Then the disease spread to almost every province of China, and 613485 AHC cases were reported from 2004 to 2014^9^. Although the case fatality of the disease is low, the number of reported cases is high, and therefore it can affect human health worldwide.

Mathematical models, including agent-based model and ordinary differential equation model, have been adopted to simulate the transmission of the disease or the assessment of the effectiveness of countermeasures^10–14^. In our previous studies^11–13^, the transmissibility of AHC and the effectiveness of countermeasures were estimated by employed several small-scale outbreaks in a limited area. However, the transmissibility of AHC remains unclear.

In this study, we built an AHC outbreak dataset (including 34 small-scale outbreaks in schools) in Hunan Province (a large province includes 14 cities and 122 counties and has a population of more than 68 million; counties were included in cities based on Chinese geography), central China from January 2007 to December 2016. The outbreaks occurred at different schools across the cities and counties. According to our previous studies^11–13^, the effective reproduction number (*R*_*eff*_), which is defined as the average number of secondary infections caused by a single infected person during his/her entire infectious period, was employed to quantify the transmissibility of AHC. In each small-scale outbreak at school, the epidemic curve was divided into two parts (uncontrolled part and controlled part) according to the date of the outbreak reported to the local public health department and intervention implemented. Consequently, *R*_*eff*_ was divided into two parts (*R*_*unc*_ and *R*_*con*_) where *R*_*unc*_ and *R*_*con*_ represent the uncontrolled and controlled *R*_*eff*_, respectively. A Susceptible-Infectious-Recovered (SIR) model was adopted to calculate the *R*_*eff*_ of AHC in each outbreak.

## Results

### Epidemiological features

**Figure 1:**
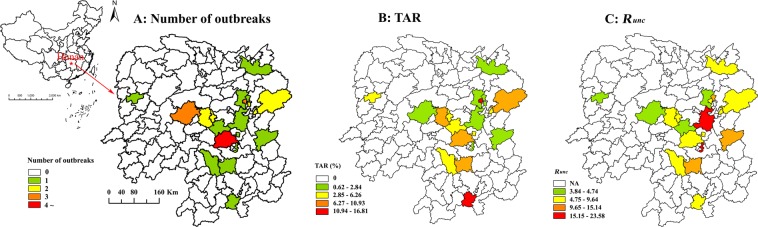
Spatial distribution of 34 reported AHC outbreaks, TAR, and *R*_*unc*_ in Hunan Province. (**A**) Number of outbreaks; (**B**) mean value of TAR in each county; (**C**) mean value of *R*_*unc*_ in each county.

**Figure 2:**
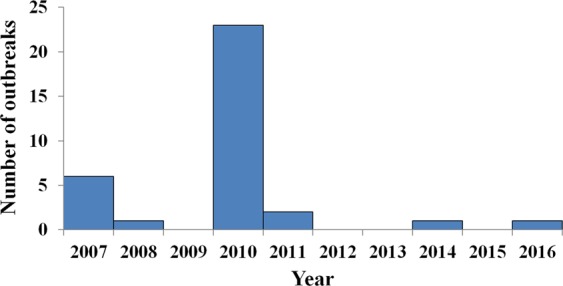
Temporal distribution of 34 AHC outbreaks in Hunan Province from 2007 to 2016.

From January 2007 to December 2016, 34 AHC outbreaks were reported in 20 counties of 9 cities in Hunan Province (Fig. 1A). 67.65% (23/34) of them occurred in 2010, 17.65% (6/34) occurred in 2007, and the others were reported in 2008, 2011, 2014, and 2016, respectively (Fig. 2).

The mean total attack rate (TAR), which is defined as the number of new cases in the population at risk divided by the number of persons at risk in the population (affected population), of the 34 outbreaks was 7.04% (95% CI: 4.97–9.11%), with the lowest one in 2008 and highest one in 2014 (Table 1). No death case was reported in the 34 outbreaks. 94.12% (32/34) of the outbreak occurred from July to September which showed an apparent seasonality. Hengyang City and Changsha City had the highest number of the outbreak, followed by Loudi City and Zhuzhou City. Two places (Yuelu District in Changsha City and Linwu County in Chenzhou City) had the highest TAR (Fig. 1B).

**Table 1:** TAR, *R*_*unc*_ and their potential risk factors in 34 AHC outbreaks in Hunan Province, China.

	TAR (%)				*R*_*unc*_		
	N	Mean	95% CI		N	Mean	95% CI
Pooled	34	7.04	4.97–9.11		32	8.28	6.46–10.11
Year							
2007	6	1.87	0.30–3.45		5	12.08	−0.52–24.68
2008	1	0.94	NA		0	NA	NA
2010	23	8.04	5.43–10.65		23	7.42	6.02–8.82
2011	2	8.98	−53.86–71.81		2	7.39	2.10–12.68
2014	1	15.35	NA	.	1	4.12	NA
2016	1	9.07	NA	.	1	15.14	NA
Seasons							
1 (January–March)	1	0.94	NA		0	NA	NA
2 (April–June)	1	9.07	NA		1	15.14	NA
3 (July–September)	32	7.17	5.00–9.34		31	8.06	6.23–9.90
4 (October–December)	0	NA	NA		0	NA	NA
City							
Chenzhou	1	15.10	NA	.	1	8.15	NA
Hengyang	9	8.97	4.23–13.70		9	9.76	4.57–14.96
Loudi	8	4.65	1.48–7.82		8	5.16	3.50–6.82
Xiangtan	1	0.61	NA	.	1	21.74	NA
Xiangxi	1	4.49	NA	.	1	3.52	NA
Yongzhou	1	5.72	NA	.	1	6.68	NA
Yueyang	1	1.24	NA	.	1	6.94	NA
Changsha	9	8.82	3.05–14.58		7	8.49	5.48–11.51
Zhuzhou	3	5.02	−6.20–16.24		3	9.82	0.66–18.98
Rural vs Urban							
Rural	18	6.93	4.40–9.45		18	7.27	5.48–9.06
Urban	16	7.17	3.94–10.41		14	9.59	6.34–12.84
Categories of school							
College	3	5.54	−5.00–16.08		3	11.49	−14.94–37.93
Secondary	18	6.42	3.63–9.21		18	8.72	6.40–11.04
Primary + Secondary	3	7.00	−11.57–25.56	.	1	4.12	.
Primary	10	8.63	3.62–13.64		10	6.95	4.21–9.69
Population of school							
0–999	9	9.73	4.53–14.93		9	9.83	4.76–14.91
1000–1999	12	9.23	5.79–12.66		12	8.61	6.74–10.48
2000–2999	7	4.75	0.87–8.63		7	6.08	4.72–7.44
>=3000	6	1.32	−0.43–3.07		4	7.67	−7.76–23.10
Number of index cases							
1	21	6.11	3.89–8.32		21	8.76	6.55–10.96
2	2	6.72	−70.91–84.35		2	14.06	−83.60–111.71
3	2	18.74	−27.45–64.92		2	7.03	−7.17–21.23
>=4	7	7.80	1.83–13.78		7	5.58	2.19–8.96

TAR, total attack rate; CI, confidence interval, the 95% CIs of TAR were calculated by binomial distribution method and those of Runc were calculated by t distribution method which were all performed by SPSS 13.0; NA, not available.

The TARs in rural areas were similar to the rates in urban areas. The difference between the TARs in rural areas and the ones in urban areas was not significant by *t*-test (*t* = −0.120, *P* = 0.905). Tested by analysis of variance (ANOVA), the differences of TARs among college, secondary, and primary schools were not significant (*F* = 0.347, *P* = 0.792), neither. However, different levels of school population had different TARs (*F* = 4.401, *P* = 0.011). Compared by least significant difference (LSD) method, the TARs of schools that had population level of “≥3000” was different to the population level of “0–999” (*P* = 0.004) and population level of “1000–1999” (*P* = 0.005) (Table 2). After running in SPSS 13.0, the 11 equations (Linear, Logarithmic, Inverse, Quadratic, Cubic, Compound, Power, S, Growth, Exponential, Logistic) fitted the relationship between TAR and population significantly. The equations Compound, Growth, Exponential, and Logistic had the highest *R*^2^, and presented a descending trend and were overlapped (Supplementary Fig. 1).

**Table 2:** Differences of TAR between any two population levels by LSD method.

	0–999	1000–1999	2000–2999	>=3000
0–999	0.000			
1000–1999	0.503	0.000		
2000–2999	4.980	4.477	0.000	
>=3000	8.410*	7.907*	3.430	0.000

*P < 0.05.

Different number of index cases also had different TARs (*F* = 3.325, *P* = 0.034). Compared by LSD method, the TARs of schools that had 3 index cases were different to that of schools with only 1 index case (*P* = 0.004), 2 index cases (*P* = 0.035), and ≥4 index cases (*P* = 0.018) (Table 3). The 11 equations could not fit the relationship between TAR and index cases significantly. Four equations (Logarithmic, Inverse, Power, and S) fitted the relationship between TAR and incidence on the first day (*I*_*fd*_) significantly, and showed the increasing trend (Supplementary Fig. 2).

**Table 3:** Differences of TAR between any two levels of index cases by LSD method.

	1	2	3	>=4
1	0.000			
2	−0.611	0.000		
3	−12.626*	−12.015*	0.000	
>=4	−1.696	−1.084	10.931*	0.000

*P < 0.05.

Teacher and staff cases were occurred in 6 outbreaks (Table 4). The differences of TARs were significant between student population and teacher and staff population in three outbreaks. The TAR of teacher and staff was higher than that of the student population in one outbreak, and the opposite results of the relationship were observed in two outbreaks. No family members or friends of the reported cases were reported infected.

**Table 4:** Differences of TARs between student population and teacher and staff population.

Outbreak ID	TAR in student population			TAR in teacher and staff population			*χ*^2^	*P*
	Affected population	Number of cases	TAR (%)	Affected population	Number of cases	TAR (%)		
13	1069	172	16.09	71	3	4.23	7.212	0.007
16	939	67	7.14	64	1	1.56	2.128	0.145
17	2169	176	8.11	62	2	3.23	1.353	0.245
19	456	53	11.62	50	1	2.00	4.377	0.036
23	443	24	5.42	20	5	25.00	9.386	0.002
33	786	13	1.65	100	4	4.00	1.498	0.221

### Transmissibility of AHC

**Figure 3:**
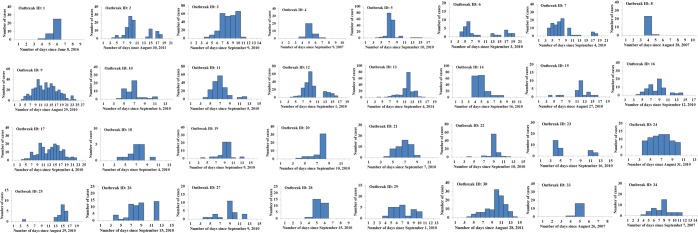
The epidemic curves of 32 outbreaks selected for calculating *R*_*unc*_ and *R*_*con*_ in Hunan Province, China.

The data of 32 outbreaks happened at schools were available for calculating *R*_*unc*_. The epidemic curves of the 32 outbreaks were shown in Fig. 3 and Supplementary Data. The mean *R*_*unc*_ of the 32 outbreaks was 8.28 (95% CI: 6.46–10.11), with the lowest mean in 2014 and the highest mean in 2016 (Table 1). Among the 32 outbreaks, 31 were occurred from July to September with mean *R*_*unc*_ of 8.06 (95% CI: 6.23–9.90). Xiangtan City had the highest *R*_*unc*_, followed by Zhuzhou City and Hengyang City. Two counties or districts (Zhuhui District in Hengyang City and Xiangtan County in Xiangtan City) had the highest *R*_*unc*_ (Fig. 1C).

The mean *R*_*unc*_ in rural areas were similar to the ones in urban areas, of which the difference was not significant by *t* test (*t* = −1.296, *P* = 0.205). Tested by ANOVA, the differences of *R*_*unc*_ among college, secondary, and primary schools were not statistically significant (*F* = 0.890, *P* = 0.459). Different levels of school population did not have different *R*_*unc*_ (*F* = 0.738, *P* = 0.538). Run in SPSS 13.0, only Cubic equation fitted the relationship between *R*_*unc*_ and school population significantly (Supplementary Fig. 3).

Different number of index cases also did not have different *R*_*unc*_ (*F* = 1.749, *P* = 0.180). Two equations (Power and S) fitted the relationship between *R*_*unc*_ and index cases significantly, however equation Power showed the decreasing trend more reasonably than S (Supplementary Fig. 4). All the 11 equations could not fit the relationship between *R*_*unc*_ and *I*_*fd*_ significantly.

### Implemented countermeasures and *R*_*con*_ of AHC

Case isolation, treatment, and health education were implemented after all the outbreaks. Environment disinfection was implemented in 33 outbreaks, symptom surveillance in 15 outbreaks, school closure in 11 outbreaks, social distance increase in 6 outbreaks, and prophylaxis and stopping eye exercises temporary in 3 outbreaks (Table 5). *R*_*con*_ and index of the effectiveness of countermeasures (*I*_*eff*_) were calculated in 32 outbreaks. The mean value of *R*_*con*_ was 0.16 (range: 0.00–1.50). The mean value of *I*_*eff*_ was 97.16% (range: 71.44–100.00%).

**Table 5:** Countermeasures and their effectiveness in each outbreak in Hunan Province, China.

Outbreak ID	Year	Month	*R*_*con*_	*I*_*eff*_ (%)	Isolation	Treatment	School closure	Environment disinfection	Health education	Symptom surveillance	Social distance	Prophylaxis	Stopping eye health exercises temporary
1	2016	6	0.00	100.00	Yes	Yes	Yes	Yes	Yes	No	Yes	No	No
2	2011	9	0.43	94.45	Yes	Yes	Yes	Yes	Yes	Yes	No	No	No
3	2010	9	0.04	99.48	Yes	Yes	Yes	Yes	Yes	No	No	No	No
4	2007	9	0.05	99.75	Yes	Yes	No	Yes	Yes	No	No	Yes	No
5	2010	9	0.17	98.78	Yes	Yes	Yes	Yes	Yes	No	No	No	No
6	2010	9	0.58	90.23	Yes	Yes	No	Yes	Yes	No	No	No	Yes
7	2010	9	0.43	95.08	Yes	Yes	Yes	Yes	Yes	No	No	No	Yes
8	2007	8	0.00	100.00	Yes	Yes	No	Yes	Yes	No	No	No	No
9	2010	9	0.11	93.24	Yes	Yes	No	No	Yes	No	No	No	No
10	2010	9	0.07	99.42	Yes	Yes	No	Yes	Yes	Yes	No	No	No
11	2010	9	0.13	98.80	Yes	Yes	No	Yes	Yes	Yes	No	No	No
12	2010	9	0.17	97.89	Yes	Yes	No	Yes	Yes	Yes	No	No	No
13	2014	9	0.11	98.82	Yes	Yes	Yes	Yes	Yes	Yes	No	No	No
14	2010	9	0.06	99.24	Yes	Yes	No	Yes	Yes	Yes	No	No	No
15	2010	9	0.13	96.22	Yes	Yes	No	Yes	Yes	No	No	No	No
16	2010	9	0.29	96.74	Yes	Yes	No	Yes	Yes	No	No	No	No
17	2010	9	1.50*	71.44	Yes	Yes	No	Yes	Yes	No	No	No	No
18	2010	9	0.00	100.00	Yes	Yes	No	Yes	Yes	No	Yes	No	No
19	2010	9	0.00	100.00	Yes	Yes	No	Yes	Yes	No	No	No	No
20	2010	9	0.00	100.00	Yes	Yes	Yes	Yes	Yes	No	No	No	No
21	2010	9	0.07	99.35	Yes	Yes	No	Yes	Yes	Yes	No	No	No
22	2010	9	0.00	100.00	Yes	Yes	No	Yes	Yes	Yes	Yes	No	No
23	2010	9	0.33	90.40	Yes	Yes	Yes	Yes	Yes	Yes	No	No	No
24	2010	9	0.08	98.35	Yes	Yes	No	Yes	Yes	Yes	Yes	No	Yes
25	2010	9	0.09	96.77	Yes	Yes	Yes	Yes	Yes	Yes	Yes	No	No
26	2010	9	0.00	100.00	Yes	Yes	Yes	Yes	Yes	Yes	No	No	No
27	2010	9	0.00	100.00	Yes	Yes	Yes	Yes	Yes	Yes	No	No	No
28	2010	9	0.00	100.00	Yes	Yes	No	Yes	Yes	No	No	No	No
29	2007	9	0.08	98.90	Yes	Yes	No	Yes	Yes	No	No	No	No
30	2011	8	0.21	97.04	Yes	Yes	No	Yes	Yes	No	No	No	No
31	2007	8	NA	NA	Yes	Yes	No	Yes	Yes	Yes	No	Yes	No
32	2008	3	NA	NA	Yes	Yes	No	Yes	Yes	Yes	No	Yes	No
33	2007	8	0.00	100.00	Yes	Yes	No	Yes	Yes	No	No	No	No
34	2007	9	0.10	98.71	Yes	Yes	No	Yes	Yes	No	Yes	No	No

NA, not available; *average value of 2.71 and 0.29.

### Sensitivity analysis

Considering that parameter *γ* in SIR model was from the published references, sensitivity analysis was performed by changing the parameter in four randomly selected outbreaks (outbreak ID: 9, 11, 16, and 30). Our model is only slightly sensitive to the parameter, the value which we set in our model (*γ* = 0.125) lead to almost the same prevalence to the mean value, mean − sd, and mean + sd of sensitivity analysis based on the 1,000 of the model ran (Supplementary Fig. 5).

## Discussion

In this study, we found that small-scale outbreaks of AHC occurred commonly in primary and secondary schools, and even in college. Due to the more frequent contact of individuals in school^15^, the transmissibility of AHC in school might be higher than the ones in the community. In our study areas, there was no outbreak reported in the community. Most of the outbreaks were occurred in primary and secondary schools from July to September, especially in September. The transmission mechanism of the outbreak remains unknown. However, we could assume that one or two students were infected by the virus during summer vacation and brought the virus back to school, leading to the transmission occurred due to the high frequent contact in school. Therefore, to prevent the transmission and make the interventions more effective, we recommended that prevention measures (including surveillance and hand hygiene) should be enhanced at the beginning of the school term in September.

By performing case-finding procedure which covered all the persons at risk (students, teachers and staffs in schools, and the family members or friends of the reported cases), cases were reported mostly among students, occasionally among teachers and staffs in schools, and none among the family members or friends. The reasons of the findings might be that: (1) the contact frequency among the students was higher than the other populations; (2) the heterogeneity of TARs existed among the different populations; (3) the adults might pay more attention about hand hygiene than children; (4) the hand hygiene of the family members might be performed more frequently during the outbreak than that in daily life; (5) hand hygiene in families was easier to implement than in schools. Therefore, more investigations were needed to explore the reasons.

TAR was similar in rural and urban areas, and different categories of school. However, it was different among the four levels of the school population, and among the four levels of index cases. The school which had a smaller population and a higher number of index cases (more than three cases) had smaller TAR. This significance might be due to the definition of TAR, and the finding of the relationship between TAR and index case is interesting and help control the disease. However, according to the results of LSD test, the TAR resulted from 3 index cases is different from those resulting from all the other index case levels. This finding might be due to the small sample size of the outbreaks which had 2 or 3 index cases (Table 1). Therefore, large data is needed to quantify the relationship between TAR and the number of index cases.

The transmissibility of AHC is high in school population and is similar to the influenza virus in small-scale outbreaks calculated by Chen *et al*.^16^. The unbalanced spatial distribution of *R*_*unc*_ was observed among different counties in Hunan Province. These indicate that area-specific countermeasures should be implemented among the high transmission areas. Although the transmissibility was not significant between rural and urban areas, among four categories of schools, among four levels of population, and among four levels of index cases, Cubic equation fits *R*_*unc*_ well with school population. Power and S equations fit *R*_*unc*_ well with number of index cases. The reasons of these finding remain unclear. It might be resulted from the limited number of outbreaks which led to some outliers (Supplementary Figs. 3 and 4). Therefore, large data is needed to quantify the relationships accurately.

Our study showed that case isolation, treatment, environmental disinfection, and health education were commonly implemented to control the outbreaks, and the other countermeasures (including surveillance, school closure, social distance, etc.) were also employed occasionally. The effectiveness of countermeasures was satisfying (higher than 90.00%) in most outbreaks, except for the *I*_*eff*_ of 71.44% in one outbreak. However, the effectiveness of countermeasures was mixed. It would be more helpful for the primary public health providers to choose an optimized AHC control strategy if the effectiveness of a specific intervention is assessed and a priority list of countermeasures is provided.

Of note, there is a limitation in our study that we only collected 34 AHC outbreaks in Hunan Province. We believe that the number of outbreaks is large enough for calculating the transmissibility of AHC, but is not large enough to analyze the relationship between *R*_*unc*_ and its risk factors. Therefore, more data should be collected to investigate the characteristics of AHC’s transmissibility, thus to control the outbreak more specifically. The second limitation is that the decrease in *R*_*eff*_ might not totally be due to the interventions. There are many other features (depletion of susceptibles, general awareness of population, etc.) that could be the reason for the decrease in *R*_*eff*_. Therefore, according to the definition of *R*_*unc*_ and *R*_*con*_, the effectiveness of the interventions might be overestimated by using the index *I*_*eff*_. Another limitation is that the incubation period was not considered in the model although AHC has a short incubation period^17^. A short incubation period might lead to a short delay of the interventions, and might affect the estimation of *R*_*con*_ slightly.

## Materials and Methods

### Ethics statement

This effort of outbreak control and investigation was part of CDC’s routine responsibility in Hunan Province; therefore, institutional review and informed consent were waived by Medical Ethics Committee of Hunan Provincial Center for Disease Control and Prevention on the following grounds: (1) only broad information about the date of the outbreaks occurred, the number of cases per day during the outbreak period, the number of affected population (students, teacher and staffs) with no identifying patient information; (2) neither medical intervention nor biological samples were involved; (3) study procedures and results would not affect clinical management of patients in any form.

### Data collection

In this study, an AHC outbreak dataset was built in Hunan Province. The dataset was collected through the Information System for Public Health Emergencies (ISPHE) from January 2007 to December 2016. AHC outbreaks were reported through the following ways: (a) reported by schools; (b) reported by hospitals, clinics or primary health care centers; and c) detected by local CDC through scanning the AHC cases reported from local hospitals, clinics or primary health care centers. The outbreak, which had 20 cases during a week, was reported to the ISPHE system. AHC cases were diagnosed according to the “Diagnosis Criteria for Acute Hemorrhagic Conjunctivitis (WS 217-2001 and WS 217-2008)” announced by the National Health Commission of the People’s Republic of China. In each outbreak, case-finding was performed according to the case definition based on the diagnostic criteria. Case-finding procedure covered the affected (potential infected) population including all the persons in school and the family members of the cases.

The dataset included the outbreak date, illness onset date of each case, number of the index cases, total outbreak cases, outbreak location, category of the affected school, the population of the school, date of countermeasures (symptom surveillance, isolation, treatment, prophylaxis, environment disinfection, social distance, health education, stopping eye health exercises temporary, class/grade/school closure) implemented, and duration of class/grade/school closure.

Symptom surveillance on “red eyes” was implemented every day from the reported outbreak date to the end of the outbreak. For case isolation, infected individuals were isolated at home until all the symptoms disappeared after 48 h. For treatment, cases were treated by medication to relieve the symptoms in the hospital or at home. Ribavirin eye drops and chloramphenicol eye drops were used for prophylaxis.

Local public health providers also disinfected the potential environment contaminated by cases, and a chlorine-based disinfectant was employed to sterilize the fomites. People were asked to keep social distance and stop eye exercises temporary during the outbreak. Local CDC staff had overseen health education for the affected people who were taught to maintain personal hygiene during the outbreak.

In order to protect young people’s eyesight, most schools in China regularly (twice per day) carry out eye exercises during the break. Eye health exercise is a kind of eye health gymnastics, mainly through massaging eye acupoints, adjusting the blood circulation of eyes and head, regulating muscles, improving eye fatigue, preventing myopia and other eye diseases. During the AHC outbreak, the eye health exercises were stopped temporarily until the transmission was completely controlled.

During the period of the class/grade/school closure, a teacher was in charge of monitoring all the students in the class every day. At the same time, each student case was asked to stay at home, observed their eyes every day and reported their findings to their teacher by telephone.

### Calculation of the transmissibility

The transmissibility of AHC was estimated by using effective reproduction number (*R*_*eff*_) which was defined as the average number of secondary infections that arise from a typical original case^18,19^. In this study, we calculated the reproduction number without control measures (*R*_*unc*_) and with control measures (*R*_*con*_). The value of *R*_*eff*_ was calculated according to the equation as follows:

$${R}_{eff}=\frac{\beta S}{\gamma }$$

In the equation, *S*, *β*, and *γ* refer to susceptible individuals, transmission rate, and recovery rate, respectively. The parameter *β* was calculated using curve fitting procedure by a SIR model employed in our published articles^11–13^. The model based on the following equations:

$$\{\begin{array}{c}\frac{dS}{dt}=-\,\beta SI\\ \frac{dI}{dt}=\beta SI-\gamma I\\ \frac{dR}{dt}=\gamma I\end{array}$$

In the model, *S*, *I*, and *R* refer to susceptible, infectious, and recovered individuals, respectively. For AHC, the infectious period is 7–10 days, 8 days were selected as the average infectious period, thus *γ* was 0.125 in the model^13^.

### Sensitivity analysis

Considering that parameter *γ* in SIR model was from the published references^13^, uncertainty might exist for our simulation results. Thus, we did a sensitivity analysis by changing the values of the parameter. During the process, the theoretical range of the parameter was split into 1000 values based on the epidemiological characteristics of AHC from 7 to 10 days for infectious period^13^. Four outbreaks (12.5% of the 32 outbreaks which had epidemic curve data) were selected randomly to run sensitivity analysis (Supplementary Fig. 5).

### Simulation and statistical methods

**Figure 4:**
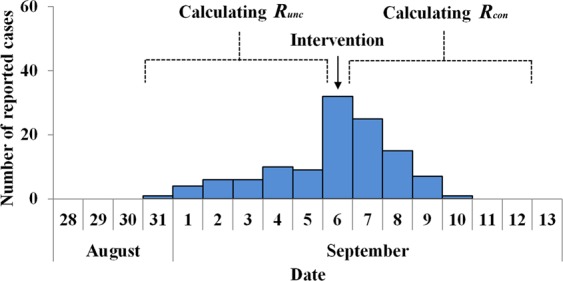
The example for curve fitting to calculate *R*_*unc*_ and *R*_*con*_ using in a small-scale outbreak in school. In this example, epidemic curve was divided into two parts (without and with intervention) according to the date when the interventions were implemented. The *R*_*eff*_ of AHC, which was denoted as *R*_*unc*_ during the part without intervention and was denoted as *R*_*con*_ after the interventions implemented, was assumed to be different between the two parts.

The SIR model was employed to fit the epidemic curve of each outbreak. At the curve fitting step, epidemic curve was divided into two parts (without and with intervention) according to the date when the interventions were implemented. We assumed that the transmissibility of AHC was different between the two parts. We defined *β* without and with control measures as *β*_*unc*_ and*β*_*con*_, respectively. And finally the *R*_*eff*_ was estimated denoted as *R*_*unc*_ during the part without intervention and was denoted as *R*_*con*_ after the interventions implemented (Fig. 4). An index of effectiveness of countermeasures (*I*_*eff*_) was developed to assess the effectiveness of countermeasures in each outbreak and was calculated by the equation as follows:

$${I}_{eff}=\frac{{R}_{unc}-{R}_{con}}{{R}_{unc}}\times 100 \% $$

Berkeley Madonna 8.3.18 was employed for the curve fitting. The fourth-order Runge–Kutta method, with tolerance set at 0.001, was used to perform curve fitting. While the curve fitting is in progress, Berkeley Madonna displays the root mean square deviation between the data and best run so far.

SPSS 13.0 was employed to run the *t*-test, ANOVA, and curve fitting of 11 equations (Linear, Logarithmic, Inverse, Quadratic, Cubic, Compound, Power, S, Growth, Exponential, Logistic) to estimate the relationship between any dependent variables (TAR and *R*_*unc*_) and independent variables (affected population, number of index cases, and incidence on the first day). The equations of the 11 models were shown as follows:

$${\rm{Linear}}:f(x)={b}_{0}+{b}_{1}x$$

$${\rm{Logarithmic}}:f(x)={b}_{0}+{b}_{1}\,\mathrm{ln}\,(x)$$

$${\rm{Inverse}}:f(x)={b}_{0}+\frac{{b}_{1}}{x}$$

$${\rm{Quadratic}}:f(x)={b}_{0}+{b}_{1}x+{b}_{2}{x}^{2}$$

$${\rm{Cubic}}:f(x)={b}_{0}+{b}_{1}x+{b}_{2}{x}^{2}+{b}_{3}{x}^{3}$$

$${\rm{Compound}}:f(x)={b}_{0}+{b}_{1}^{x}$$

$${\rm{Power}}:f(x)={b}_{0}+{x}^{{b}_{1}}$$

$${\rm{S}}:f(x)={e}^{({b}_{0}+\frac{{b}_{1}}{x})}$$

$${\rm{Growth}}:f(x)={e}^{({b}_{0}+{b}_{1}x)}$$

$${\rm{Exponential}}:f(x)={b}_{0}{e}^{{b}_{1}x}$$

$${\rm{Logistic}}:f(x)=\frac{1}{\frac{1}{u}+{b}_{0}+{b}_{1}^{x}}$$

In the equations, *x* and *f*(*x*) refer to the independent (affected population, number of index cases, and incidence on the first day) and dependent variables (total attack rate and *R*_*unc*_), repectively; *b*_0_, *b*_1_, *b*_2_, *b*_3_, and *u* refer to the coefficients of the models which were estimated by curve fitting with the data.

Determination coefficient (*R*^2^) was employed to evaluate the curve fitting. Total attack rate (TAR) and incidence on the first day (*I*_*fd*_) were calculated by the following equations:

$$TAR=\frac{{N}_{A}}{{N}_{p}}\times 100 \% $$

$${I}_{fd}=\frac{{N}_{ic}}{{N}_{p}}\times 100 \% $$

*N*_*A*_, *N*_*ic*_, and *N*_*p*_ refer to the number of total cases in the outbreak, the number of index cases, and population of the school, respectively.

The 95% CIs of TAR were calculated by binomial distribution method and those of *R*_*unc*_ were calculated by *t* distribution method. These procedures were all performed by SPSS 13.0.

## Supplementary information

Supplementary Information 

Supplementary Information 2 
