# Supplementary material for: Transmissibility of acute haemorrhagic conjunctivitis in small-scale outbreaks in Hunan Province, China
Source: Sci Rep. 2020 Jan 10;10:119. doi: 10.1038/s41598-019-56850-9 (PMC6954223; doi:10.1038/s41598-019-56850-9)
Supplement: Supplementary file 1 — Supplementary Information [file 41598_2019_56850_MOESM1_ESM.docx]

**Supplementary Figures**

**Manuscript title:**

**Transmissibility of acute haemorrhagic conjunctivitis in small-scale outbreaks in Hunan Province, China**

**List of authors:**

**Siyu Zhang^1*^, Qingqing Hu^2*^, Zhihong Deng^1^, Shixiong Hu^1^, Fuqiang Liu^1^, Shanshan Yu^3^, Ruoyun Liu^3^, Chunlei He^3^, Hongye Li^3^, Lidong Gao^1∆^, Tianmu Chen^3∆#^**


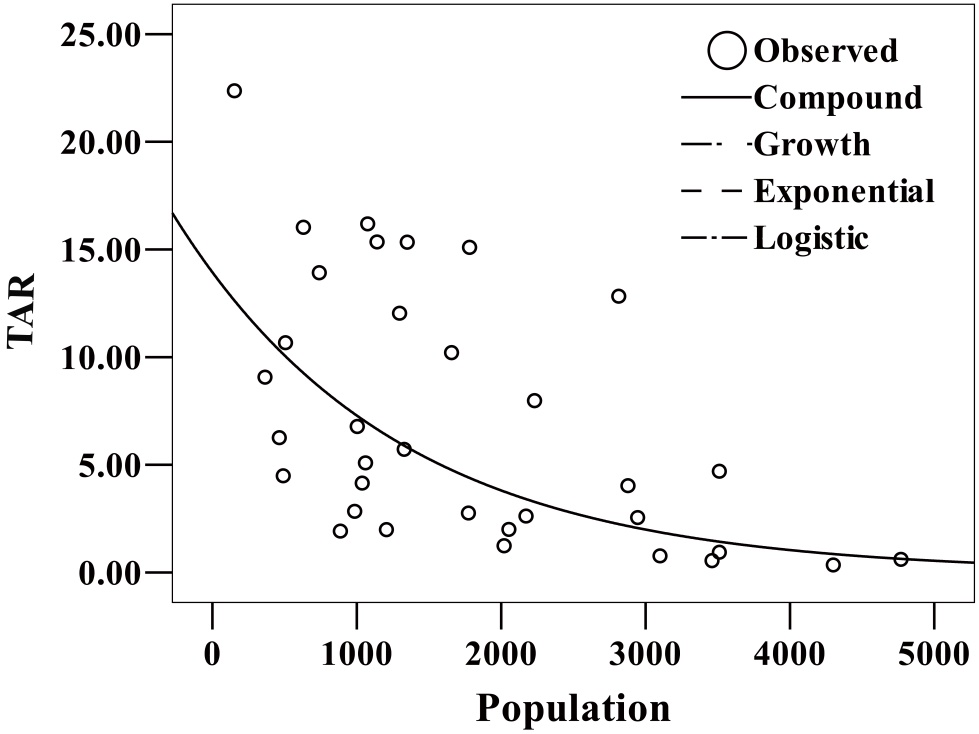


**Supplementary Figure 1. The best fitted models (Compound, Growth, Exponential, and Logistic equations) to show the relationship between TAR and population.** The lines of the four equations overlapped.


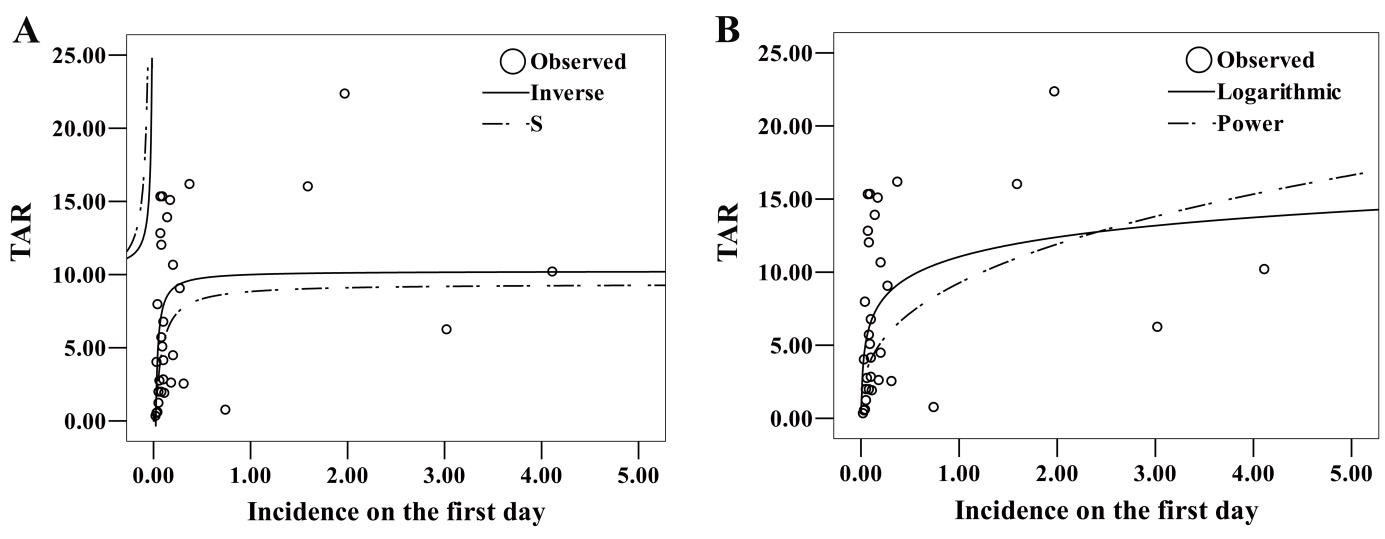


**Supplementary Figure 2. The best fitted models (Inverse, S, Logarithmic, and Power equations) to show the relationship between TAR and incidence on the first day.** A, fitted by Inverse and S equation; B, fitted by Logarithmic and Power equation.


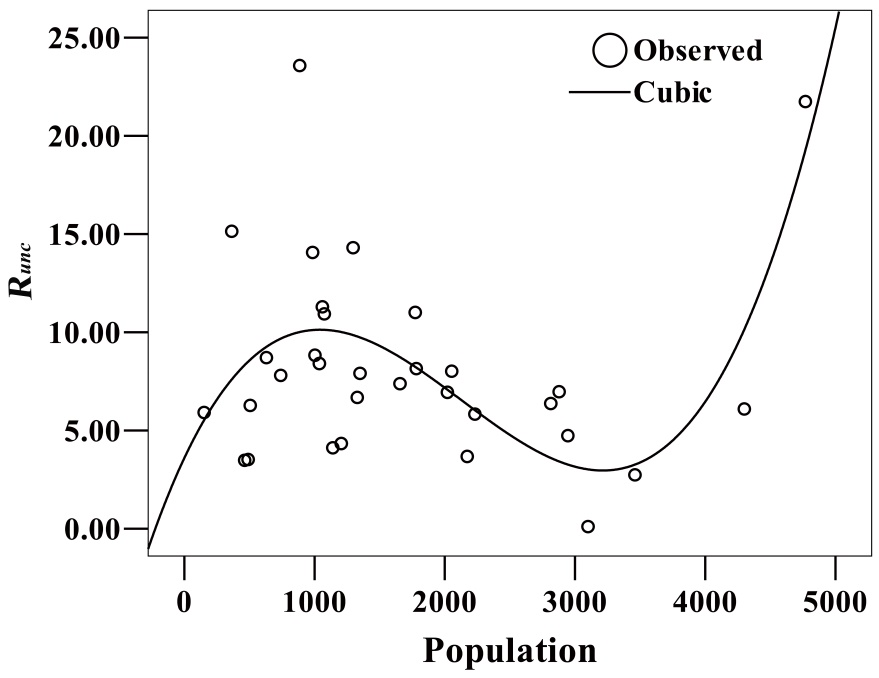


**Supplementary Figure 3. The relationship between *R_unc_* and population fitted by Cubic equation.**

**
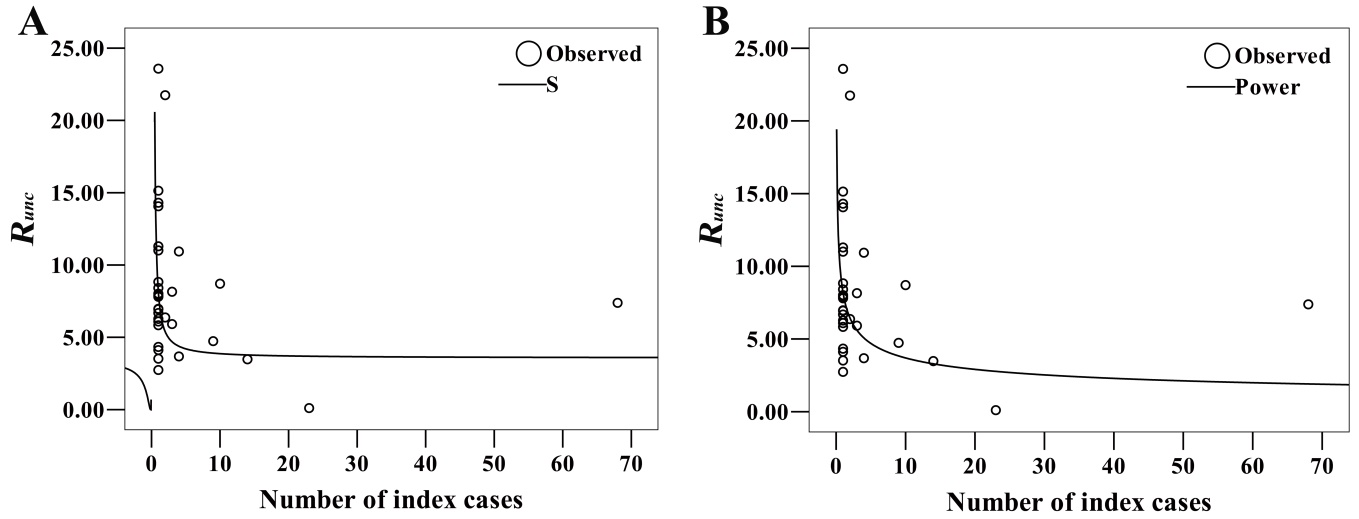
**

**Supplementary Figure 4. The relationship between *R_unc_* and index cases fitted by S and Power equation.** A, fitted by S equation; B, fitted by Power equation.


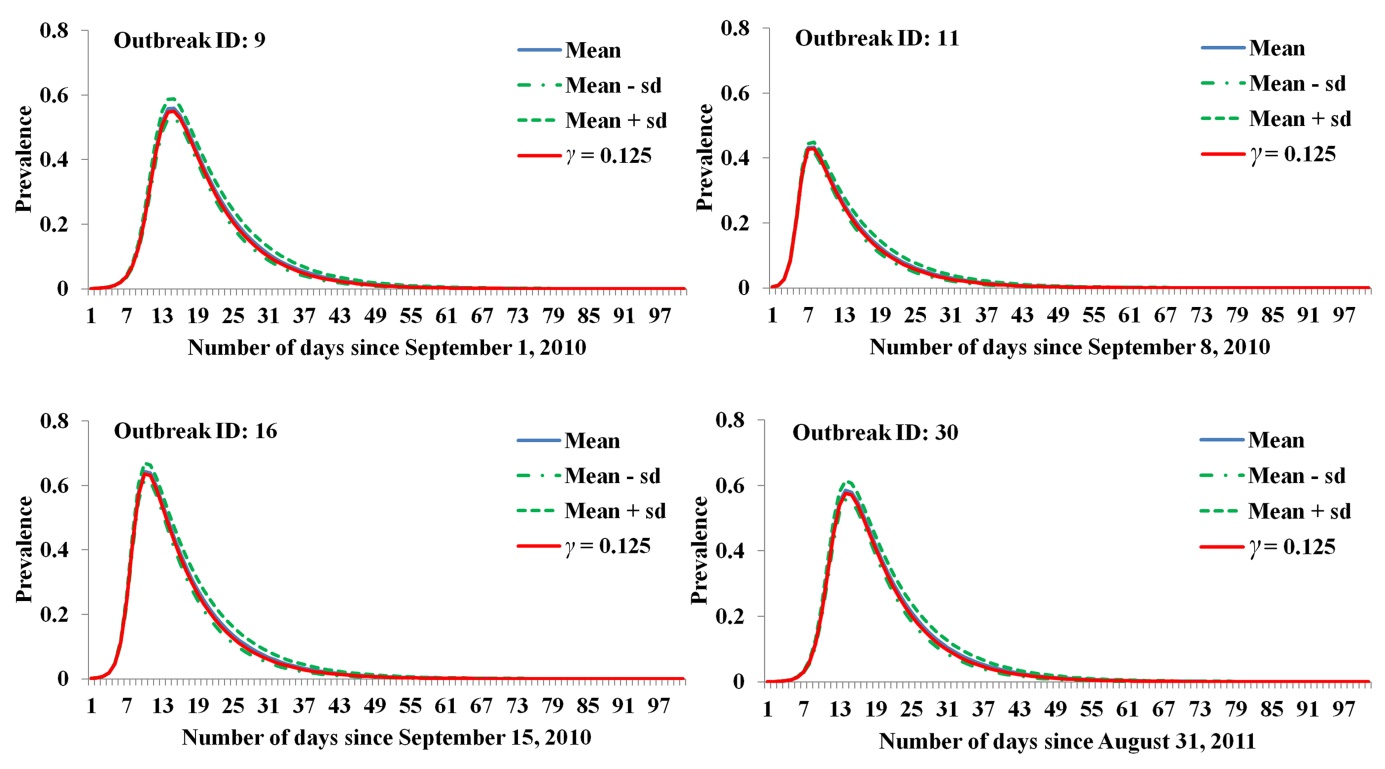


**Supplementary Figure 5. Sensitivity analysis performed by changing the parameter** ***γ* in four randomly selected outbreaks.**
